# Clinical implications of the proposed ICD-11 PTSD diagnostic criteria

**DOI:** 10.1017/S0033291718001101

**Published:** 2018-05-14

**Authors:** Anna C. Barbano, Willem F. van der Mei, Richard A. Bryant, Douglas L. Delahanty, Terri A. deRoon-Cassini, Yutaka J. Matsuoka, Miranda Olff, Wei Qi, Andrew Ratanatharathorn, Ulrich Schnyder, Soraya Seedat, Ronald C. Kessler, Karestan C. Koenen, Arieh Y. Shalev

**Affiliations:** 1 Department of Psychiatry, New York University School of Medicine, 1 Park Avenue, New York, NY 10016, USA; 2 School of Psychology, University of New South Wales, Sydney, NSW, 2052, Australia; 3 Department of Psychological Sciences, Kent State University, 144 Kent Hall, Kent, OH 44242, USA; 4 Department of Surgery, Medical College of Wisconsin, 9200 W. Wisconsin Ave, Milwaukee, WI 53226, USA; 5 Division of Health Care Research, Center for Public Health Sciences, National Cancer Center Japan, 5-1-1 Tsukiji, Chou-ku, Tokyo 104-0045, Japan; 6 Department of Psychiatry, Academic Medical Center, University of Amsterdam, Meibergdreef 9, 1105 AZ Amsterdam, The Netherlands; 7 Arq Psychotrauma Expert Group, Postbus 240, 1110 AE, Diemen, The Netherlands; 8 Department of Epidemiology, Columbia University Mailman School of Public Health, 722 W 168th St., New York, NY 10032, USA; 9 Department of Psychiatry and Psychotherapy, University Hospital Zurich, PO Box 1931, Lenggstrasse 31, 8032, Zürich/Switzerland; 10 Department of Psychiatry, Stellenbosch University, Private Bag X1, Matieland, 7602, Stellenbosch, South Africa; 11 Department of Health Care Policy, Harvard Medical School, 180 Longwood Ave, Boston, MA 02115, USA; 12 Department of Epidemiology, Harvard T.H. Chan School of Public Health, Kresge 505, 677 Huntington Avenue, Kresge Building, Boston, MA 02115, USA

**Keywords:** Diagnosis, ICD-10, ICD-11, individual participant-level data, longitudinal, post-traumatic stress disorder

## Abstract

**Background:**

Projected changes to post-traumatic stress disorder (PTSD) diagnostic criteria in the upcoming International Classification of Diseases (ICD)-11 may affect the prevalence and severity of identified cases. This study examined differences in rates, severity, and overlap of diagnoses using ICD-10 and ICD-11 PTSD diagnostic criteria during consecutive assessments of recent survivors of traumatic events.

**Methods:**

The study sample comprised 3863 survivors of traumatic events, evaluated in 11 longitudinal studies of PTSD. ICD-10 and ICD-11 diagnostic rules were applied to the Clinician-Administered PTSD Scale (CAPS) to derive ICD-10 and ICD-11 diagnoses at different time intervals between trauma occurrence and 15 months.

**Results:**

The ICD-11 criteria identified fewer cases than the ICD-10 across assessment intervals (range −47.09% to −57.14%). Over 97% of ICD-11 PTSD cases met concurrent ICD-10 PTSD criteria. PTSD symptom severity of individuals identified by the ICD-11 criteria (CAPS total scores) was 31.38–36.49% higher than those identified by ICD-10 criteria alone. The latter, however, had CAPS scores indicative of moderate PTSD. ICD-11 was associated with similar or higher rates of comorbid mood and anxiety disorders. Individuals identified by either ICD-10 or ICD-11 shortly after traumatic events had similar longitudinal course.

**Conclusions:**

This study indicates that significantly fewer individuals would be diagnosed with PTSD using the proposed ICD-11 criteria. Though ICD-11 criteria identify more severe cases, those meeting ICD-10 but not ICD-11 criteria remain in the moderate range of PTSD symptoms. Use of ICD-11 criteria will have critical implications for case identification in clinical practice, national reporting, and research.

## Introduction

The Post-traumatic stress disorder (PTSD) diagnostic criteria have been recently revised in the Diagnostic and Statistical Manual of Mental Disorders (DSM; American Psychiatric Association, 2013), and their revision in the International Classification of Diseases (ICD; World Health Organization) is pending. The implementation of the new DSM-5 diagnostic criteria significantly affected the prevalence estimates, clinical characteristics, and overlap between samples identified as having PTSD (Hoge *et al.* 2016). The update from the 10th edition of the ICD (ICD-10; World Health Organization, 1993) to the upcoming 11th edition (ICD-11; World Health Organization, 2018) offers a particularly radical alteration. While the ICD-10 takes a fairly broad diagnostic approach and includes 13 symptoms in its diagnostic template, the ICD-11 proposes to remove the symptoms common to PTSD and other disorders (e.g. sleep disturbances, irritability) toward increasing the specificity of the diagnosis (Maercker *et al.* 2013). The proposed ICD-11 template comprises, therefore, six disorder-defining criteria: dissociative flashbacks, nightmares, hypervigilance, exaggerated startle response, avoidance of external reminders, and avoidance of thoughts and feelings associated with the traumatic event. The differences between the ICD-10 and ICD-11 diagnostic templates may significantly affect the prevalence and the clinical characteristics of patients identified, and its study is both important and timely.

Studies to date have evaluated the rates of PTSD diagnoses using ICD-10 and ICD-11 criteria among survivors of childhood institutional abuse (Knefel and Lueger-Schuster, 2013; Gluck *et al.* 2016), adult and adolescent survivors of a mass shooting (Haravuori *et al.* 2016), individuals living in conflict-torn villages in Timor-Leste (Tay *et al.* 2017), community-dwelling adults and war veterans (Wisco *et al.* 2016), and a multi-national World Mental Health survey (Stein *et al.* 2014). These studies have repeatedly shown that the ICD-11 identifies fewer PTSD cases. Most studies have also reported an overlap between ICD-10 and ICD-11 diagnoses, in which the latter identified a subset of individuals identified by the former (Gluck *et al.* 2016; Haravuori *et al.* 2016; Tay *et al.* 2017).

Studies to date have addressed chronic PTSD patients using one-time, cross-section assessment. However, a recent work has shown that PTSD symptoms fluctuate, and trauma survivors’ diagnostic statuses change following trauma exposure (Bryant *et al.* 2013). To longitudinally examine the implications of the proposed ICD revision, the current study examined ICD-10 and ICD-11 diagnoses made at different time intervals after trauma exposure. Data from 11 longitudinal studies of early PTSD in Australia, Israel, Japan, Switzerland, the USA, and the Netherlands were pooled, and ICD-10 and ICD-11 diagnoses were obtained at two time intervals after the traumatic event: 0–60 days, representing an early post-exposure period, and 122–456 days (4–15 months), representing a period of persisting PTSD symptoms. The latter was secondarily divided into a 122–270 days (4–9 months) period, during which further recovery is expected, and a 271–456 days (9–15 months) period, representing more chronic, stable PTSD. To strictly meet the ICD minimum symptom duration criterion for a PTSD diagnosis, a 22–60 days time interval was additionally used as an initial assessment time interval.

For each time interval, we compared the prevalence of PTSD per ICD-10 and ICD-11 criteria, the overlap between samples so defined, the severity of cases identified by each template, differences in age and gender distributions, the presence of comorbid mood and anxiety disorders, and the stability of both diagnostic groups over time.

## Method

### Sources of data

Data for this work were obtained from the pooled dataset of the International Consortium to Predict PTSD (ICPP; Qi *et al.* submitted for publication). ICPP studies had longitudinally evaluated adult civilians admitted to general hospitals’ emergency departments (EDs) following traumatic events that included motor vehicle accidents (71.35%), other non-interpersonal accidents (22.68%), and interpersonal violence (5.97%). Raw, item-level data shared by ICPP investigators were cleaned, harmonized, and processed to provide standardized measures of PTSD and demographic data. Eleven studies used the DSM-IV (American Psychiatric Association, 2000) version of the Clinician-Administered PTSD Scale (CAPS; Blake *et al.* 1995) and were included in the present analysis (Shalev *et al.* 2000; Bonne *et al.* 2001; Hepp *et al.* 2008; Irish *et al.* 2008; Shalev *et al.* 2008; Jenewein *et al.* 2009; Matsuoka *et al.* 2009; Shalev *et al.* 2012; Bryant *et al.* 2013; Mouthaan *et al.* 2013; Frijling *et al.* 2014) (Table 1). In total, 37.40% of the participants were females and 62.60% were males. The sample's mean age was 40.0 (s.d. = 13.93).

**Table 1.** Participating studies’ design, sample size, and follow-up periods

Study^a^	Study design	Study duration	Country	Sample size^b^ (*total*)	Sample size (*0–60 days*)	Sample size (*122–456 days*)	Trauma type (% MVA, OA, IV)^c^	Last follow-up	Number of PTSD assessments
Hepp *et al.* (2008)	Observational	1995–1997	Switzerland	121	121	109	60.9%, 39.1%, 0.0%	3 years	4
Shalev *et al.* (2000)	Observational	1995–1998	Israel	222	208	41	84.3%, 8.1%, 7.6%	4 months	2
Bonne *et al.* (2001)	Observational	1997–1998	Israel	43	0	43	79.1%, 4.6%, 16.3%	6 months	1
Jenewein *et al.* (2009)	Observational	1999–2000	Switzerland	323	323	255	30.4%, 69.6%, 0.0%	6 months	2
Irish *et al.* (2008)	Observational	2000–2004	USA	263	183	227	100.0%, 0.0%, 0.0%	1 year	3
Bryant *et al.* (2013)	Observational	2004–2006	Australia	1084	1082	828	65.1%, 29.4%, 5.5%	1 year	3
Shalev *et al.* (2008)	Observational	2005–2006	Israel	169	118	154	80.8%, 6.4%, 12.8%	4 months	2
Shalev *et al.* (2012)	Intervention	2003–2007	Israel	732	732	528	82.0%, 5.8%, 12.2%	3 years	4
Matsuoka *et al.* (2009)	Observational	2004–2008	Japan	171	155	108	100.0%, 0.0%, 0.0%	1.5 years	4
Mouthaan *et al.* (2013)	Observational	2005–2009	The Netherlands	676	478	546	70.8%, 18.8%, 10.4%	1 year	4
Frijling *et al.* (2014)^d^	Intervention	2012–2015	The Netherlands	54	54	42	65.7%, 30.5%, 3.8%	6 months	4

a Studies are listed by chronological order of the end year, from earliest to most recent studies. If two studies ended in the same year, alphabetic order of the principal investigator's last name is used.

b Sample sizes reflect the number of participants used in the current analyses (rather than the total number of participants in the original studies).

c MVA, motor vehicle accident; OA, other accident; IV, interpersonal violence.

d Unpublished at the time of data transfer.

### Instruments

The CAPS for DSM-IV, a structured clinical interview, was used to measure PTSD symptom severity and infer ICD-10 and ICD-11 diagnostic status. CAPS items include all 17 DSM-IV PTSD diagnostic criteria. It quantifies each symptom's frequency and intensity on a scale of 0–4. The CAPS total score (sum of symptoms’ intensities and frequencies, range 0–136) quantifies PTSD symptom severity. Following CAPS guidelines, we defined a symptom as positively endorsed if its frequency score was 1 or more and its intensity score 2 or more (Weathers *et al.* 1999; Weathers *et al.* 2001). The ED admission ‘index events’ were used in all subsequent PTSD evaluations.

Following previous recommendations, participants’ PTSD severity was rated as either *asymptomatic* (CAPS total scores between 0 and 19), *mild/subthreshold* (CAPS total scores between 20 and 39), *moderate* (CAPS total scores between 40 and 59), *severe* (CAPS total scores between 60 and 79), or *extreme* (CAPS total scores ⩾80) (Weathers *et al.* 1999; Blake *et al.* 2000; Weathers *et al.* 2001).

### ICD diagnoses and severity

As previously delineated (Brewin, 2013) and used (Knefel and Lueger-Schuster, 2013), ICD-10 and ICD-11 diagnoses were derived from DSM-IV symptoms aligned with the ICD-10 and ICD-11 diagnostic criteria. For ICD-10, the criteria were: one out of CAPS items 1 through 5 (B cluster); one out of items 6 and 7 (C1 and C2); and either item 8 (C3) or two out of items 13 through 17 (D cluster). For ICD-11, the criteria were: one out of CAPS items 2 and 3 (B2 and B3); one out of items 6 and 7 (C1 and C2); and one out of items 16 and 17 (D4 and D5) (Table 2). As an auxiliary analysis, we included the B1 criterion (vivid intrusive memories of the traumatic event) in the ICD-11 diagnostic formula to test an extended set of CAPS-derived ICD-11 diagnostic identifiers, in which endorsement of B1, B2, or B3 fulfilled the B criteria.

**Table 2.** CAPS items and corresponding ICD-10 and ICD-11 diagnostic criteria

CAPS item	ICD-10	ICD-11
B1. Recurrent, intrusive, distressing recollections, including images, thoughts, or perceptions of the event	X	(X)*
B2. Recurrent, distressing dreams of the event	X	X
B3. Acting or feeling as if the traumatic event were recurring (dissociative flashback episodes)	X	X
B4. Intense psychological distress at exposure to internal or external reminders of the event	X	
B5. Physiological reactivity on exposure to internal or external reminders of the event	X	
C1. Efforts to avoid thoughts, feelings, or conversations associated with the event	X	X
C2. Efforts to avoid activities, places, or people associated with the event	X	X
C3. Inability to recall an important aspect of the event	X	
C4. Markedly diminished interest or participation in significant activities		
C5. Feeling of detachment or estrangement from others		
C6. Restricted range of affect (e.g. unable to have loving feelings)		
C7. Sense of foreshortened future		
D1. Difficulty falling or staying asleep	X	
D2. Irritability or outbursts of anger	X	
D3. Difficulty concentrating	X	
D4. Hypervigilance	X	X
D5. Exaggerated startle response	X	X

*Following previous recommendations (Brewin, 2013), we used six disorder-defining symptom criteria for ICD-11 in primary analyses. However, in order to test an extended set of CAPS-derived ICD-11 diagnostic identifiers, item B1 was used in an auxiliary sensitivity analysis.

For analysis, the following groups were identified: an ‘*ICD-10*’ group (study participants who met ICD-10 diagnostic criteria), an ‘*ICD-11*’ group (participants who met ICD-11 diagnostic criteria), and a ‘*no PTSD*’ group (participants meeting neither ICD-10 nor ICD-11 diagnostic criteria). Additionally, participants who met ICD-10 but not ICD-11 criteria constituted an ‘*ICD-10-only*’ group. Precluding valid group comparisons, very few participants met ICD-11 diagnostic criteria alone (*n* < 10, and <3% at both time intervals). Patterns leading to the few ICD-11 cases not meeting ICD-10 criteria were explored.

Clinically significant impairment (CAPS criterion F) was assessed in 75.1% (*n* = 2903) of the sample and used to evaluate the effect of including this new requirement in ICD-11's diagnosis. Additionally, PTSD symptoms’ onset before 6 months (a suggested feature of ICD-10) was evaluated to ascertain the inclusion in the ICD-10 group after 6 months.

### Time intervals

Time intervals between the traumatic events and subsequent assessments were measured, for each CAPS assessment, as ‘days from trauma’. For analyses, CAPS time bracket data were grouped into two *time intervals*: an early post-exposure period extending from 0 to 60 days (first 2 months) following trauma exposure and a 122–456 days (4–15 months) time interval, during which PTSD minimum duration criteria are met. Out of 3863 participants included in this work, 3453 were present in the 0–60 days time interval, and 2880 were present in the 122–456 days time interval.

Two secondary analyses were performed: First, the 122–456 days time interval was further divided into 122–270 days (4–9 months) after trauma exposure to inform an early PTSD period, and 271–456 days (9–15 months) to inform a late, mostly stable, expression of PTSD. Second, to strictly meet the ICD minimum illness duration criterion for a PTSD diagnosis, a 22–60 time interval was additionally used for including subjects in the initial assessment.

Every participant with a CAPS assessment within a time interval was included in the analysis. The earliest CAPS evaluation was used in the participants with more than one CAPS assessment during the 0–60 and 22–60 days time intervals and the latest CAPS assessment was used during subsequent time intervals.

Participants lost to follow-up [i.e. present in the 0–60 days time interval and absent from the 122–456 days time interval (*n* = 980)] did differ from those retained in their initial PTSD symptom severity (*M* = 30.54, s.d. = 26.34 for participants lost to follow-up; *M* = 27.41, s.d. = 25.14 for participants not lost to follow-up; *p* for *t* test = 0.002).

The median times since trauma were 17 days [interquartile range (IQR) = 29] for the 0–60 days interval, 37 days (IQR = 17) for the 22–60 days interval, 321 days (IQR = 188) for the 122–456 days interval, 187 days (IQR = 50) for the 122–270 days interval, and 374 days (IQR = 33) for the 271–456 days interval.

### Concurrent psychiatric diagnoses

Concurrent DSM-IV psychiatric disorders were evaluated in 2550 (66.0%) participants using either the Mini-International Neuropsychiatric Interview (MINI; Sheehan *et al.* 1998) in three studies (Matsuoka *et al.* 2009; Bryant *et al.* 2013; Mouthaan *et al.* 2013) or the Structured Clinical Interview for DSM-IV Axis I Disorders (SCID; First *et al.* 2002) in four studies (Shalev *et al.* 2000; Bonne *et al.* 2001; Shalev *et al.* 2008; Shalev *et al.* 2012). Concurrent anxiety disorders and mood disorders (excluding bipolar disorder) were grouped to create an ‘any anxiety disorder’ variable and an ‘any depressive disorder’ variable to enable large enough numbers in each category.

### Longitudinal course

To longitudinally assess the course of early ICD-10 and ICD-11 diagnostic groups, we compared the PTSD status (recovery and migration between diagnostic groups) and symptom severity (total CAPS score) of participants originally allocated to these diagnostic categories at the 122–456 days time interval.

### Studies’ heterogeneity

In order to assess the individual studies’ contribution to the pooled data outcome, we examined heterogeneity of studies using individual participant-level data at the 0–60 and 122–456 days time intervals and combined study-by-study results.

### Data analysis

All analyses were conducted using R software (R Core Team, 2017). Means, standard deviations, and confidence intervals of CAPS scores and age were calculated for ICD classification at each time point. Proportions were calculated for gender at each time point. A χ^2^ test for proportions with a Yates continuity correction was used to test the differences in the proportions of ICD-10-only and ICD-11 diagnoses in the total sample.

Confidence intervals for gender proportions were calculated via the Score method. Welch's *t* test was used to examine the differences in mean age and CAPS total severity score between ICD-10-only and ICD-11 classifications. The all ICD-10 *v.* all ICD-11 groups were not tested, given the high degree of non-independence between the groups. Cohen's *κ* was calculated to determine the level of agreement between the diagnostic indices.

Fisher's exact test was carried out to assess the statistical significance of the relative odds of having a comorbid diagnosis of a depressive or anxiety disorder with ICD-10-only or ICD-11 PTSD for each of the SCID and the MINI.

In evaluating studies’ heterogeneity, an *I*^2^ statistic, a measure of heterogeneity in the study results, was used to determine whether a fixed- or random-effects model would be used in pooling study-by-study results. Mean differences and confidence intervals were pooled according to the inverse-variance method. Studies that did not have both ICD-10-only and ICD-11 cases were not included in this analysis.

## Results

### Cross-sectional prevalence

**Fig. 1.**
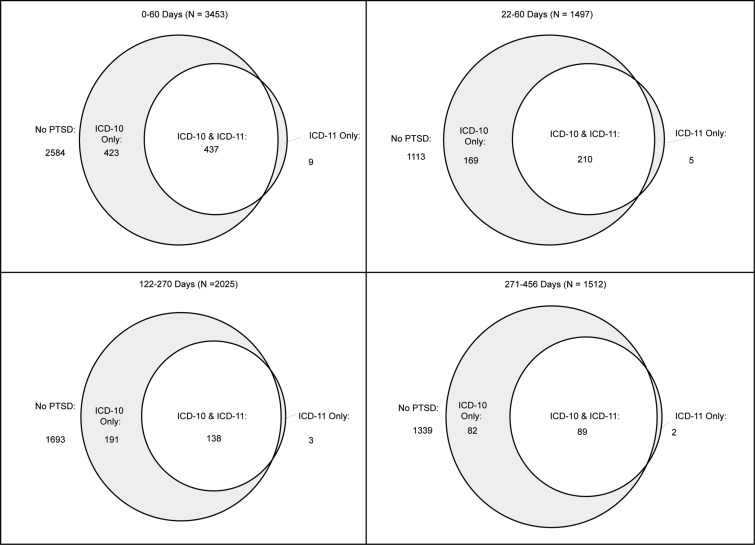
Overlap of ICD-10 and ICD-11 diagnoses at four time intervals following trauma.

During the *0–60 days time interval*, the prevalence of ICD-10 PTSD was 24.89%, and that of ICD-11 PTSD was 12.94% (Fig. 1). Most of the ICD-11 group (97.99%) also met ICD-10 diagnostic criteria. Mean CAPS scores of the participants with ICD-11 PTSD were higher than those with ICD-10 PTSD (*p* < 0.0001) (Table 3). Cohen's *κ* revealed moderate agreement between the ICD-10 and ICD-11 (*κ* = 0.60, 95% CI 0.57–0.63) (Altman, 1990).

**Table 3.** CAPS severity by PTSD group and time interval

	No PTSD mean ± s.d.	95% (CI)	ICD-10 mean ± s.d.	95% (CI)	ICD-11 mean ± s.d.	95% (CI)	ICD-10-only mean ± s.d.	95% (CI)	ICD-10-only *v.* ICD-11 *p*
0–60 Days	*N* = 2584		*N* = 860		*N* = 447		*N* = 423		
CAPS score	16.64 ± 13.70	(16.11–17.17)	63.23 ± 20.55	(61.85–64.60)	71.09 ± 20.65	(69.17–73.01)	54.11 ± 17.03	(52.48–55.73)	<0.0001
22–60 Days	*N* = 1113		*N* = 379		*N* = 216		*N* = 169		
CAPS score	17.63 ± 14.10	(16.80–18.47)	62.90 ± 20.51	(60.83–64.97)	68.83 ± 21.87	(65.90–71.78)	54.14 ± 15.99	(51.71–56.57)	<0.0001
122–456 Days	*N* = 2470		*N* = 406		*N* = 198		*N* = 212		
CAPS score	13.28 ± 13.50	(12.75–13.82)	59.26 ± 21.15	(57.19–61.32)	67.93 ± 22.27	(64.81–71.05)	50.31 ± 16.31	(48.10–52.51)	<0.0001
122–270 Days	*N* = 1693		*N* = 329		*N* = 141		*N* = 191		
CAPS score	13.34 ± 13.09	(12.72–13.97)	56.55 ± 20.83	(54.29–58.81)	65.87 ± 22.59	(62.10–69.63)	49.13 ± 16.54	(46.77–51.49)	<0.0001
271–456 Days	*N* = 1339		*N* = 172		*N* = 91		*N* = 82		
CAPS score	13.45 ± 13.35	(12.73–14.17)	62.70 ± 20.90	(59.56–65.85)	71.33 ± 21.07	(66.94–75.72)	52.26 ± 15.55	(48.84–55.67)	<0.0001

The *p* values reflect significance levels for comparisons between ICD-10-only and ICD-11 diagnoses.

During the *122–456 days time interval*, the prevalence of ICD-10 PTSD was 14.10%, and the prevalence of ICD-11 PTSD was 6.88% (Fig. 1). Most of the ICD-11 group (97.47%) also met ICD-10 diagnostic criteria. Mean CAPS scores of participants with ICD-11 PTSD were again higher than mean CAPS scores of those with ICD-10 PTSD (*p* < 0.0001) (Table 3). Cohen's *κ* revealed moderate agreement between the ICD-10 and ICD-11 (*κ* = 0.60, 95% CI 0.56–0.65) (Altman, 1990).

*Age and gender* did not differ across these time intervals between ICD-10 and ICD-11 classification. However, in comparing those who only met ICD-10 criteria with those who met ICD-11 criteria, the ICD-10-only group was associated with older age at both time intervals (for 0–60 days, *p* = 0.013; for 122–456 days, *p* = 0.013).

Results secondarily obtained during the *122–270 days* and *270–456 days time intervals* were similar in ICD-identified PTSD prevalence and difference in mean CAPS scores (Fig. 1; Table 3).

Using the *22–60 days time interval*, the prevalence of ICD-10 PTSD was 25.27% and that of ICD-11 PTSD was 14.33%. Most of the ICD-11 group (98.7%) also met ICD-10 diagnostic criteria. Mean CAPS scores of ICD-11 cases were significantly higher than mean CAPS scores of ICD-10 cases (*p* < 0.0001) (Table 3).

Including the B1 criterion in ICD-11 definition yielded somewhat higher overlap between the diagnostic groups (65.23% and 62.81% of ICD-10 cases also met ICD-11 criteria, respectively, for the early and late time intervals), similarly low prevalence of ICD-11-only cases (<3%), and higher CAPS total severity scores in the ICD-11 group at the two time intervals (*M* = 68.91, s.d. = 20.15 *v. M* = 50.77, s.d. = 16.49, *p* < 0.0001 for the early assessment; and *M* = 65.69, s.d. = 21.01 *v. M* = 46.98, s.d. = 16.03, *p* < 0.001 for the later assessment). Similar differences in symptom severity were found at the 122–270 days time interval (*p* < 0.0001) and the 271–456 days time interval (*p* < 0.0001). Importantly, the ICD-10-only group continued to express moderate-to-high levels of PTSD symptoms.

There were nine participants in the ICD-11-only group in the 0–60 days time interval and five in the 122–456 days time interval. Of these participants, none endorsed the following four ICD-10 symptom criteria: C3 (inability to recall an important aspect of the trauma), D1 (difficulty falling or staying asleep), D2 (irritability or outbursts of anger), and D3 (difficulty concentrating).

Among participants who met ICD-11 PTSD diagnostic criteria, 95.6% and 97.3% met the CAPS F criterion at the 0–60 and 122–456 days time intervals, respectively. The number of participants who did not meet the F criterion was consistently ⩽7. Based on the data, the inclusion of an F criterion would not likely significantly impact the overlap in ICD-10 and ICD-11 classifications.

There were no cases of ICD-10 PTSD onset after 6 months.

### Classification migration and trajectories

With time, the majority of both the ICD-10-only group (67.41%) and ICD-11 group (57.50%) went into remission in their respective diagnostic definition. Migration between PTSD classifications comprised 10.86% of participants with early ICD-10 classification meeting ICD-11 criteria at the later assessment and 19.64% participants with early ICD-11 classification meeting ICD-10 alone criteria at the later assessment. A small number of participants without early PTSD developed an ICD-10-only (3.72%) or an ICD-11 diagnosis (3.19%) at follow-up. Finally, 21.73% of the ICD-10-only group and 22.86% of the ICD-11 group maintained their diagnostic status over time.

### Concurrent psychiatric diagnoses

In total, 51.3% of ICD-11 cases and 39.8% of ICD-10-only cases met SCID diagnostic criteria for a depressive disorder during the 0–60 days time interval. ICD-11 cases had significantly higher odds of a SCID comorbid depressive disorder than ICD-10-only cases during the 0–60 days time interval (OR 1.59, 95% CI 1.14–2.22, *p* = 0.007), but similar odds during the 122–456 days time interval (OR 1.50, 95% CI 0.83–2.73, *p* = 0.16), in which 47.9% of ICD-11 cases and 38.0% of ICD-10-only cases met SCID diagnostic criteria for a depressive disorder. In total, 20.3% of ICD-11 cases and 20.8% of ICD-10-only cases met SCID diagnostic criteria for an anxiety disorder in the 0–60 days time interval, and the relative odds were not statistically significant (OR 0.97, 95% CI 0.64–1.46, *p* = 0.917). In total, 20.8% of ICD-11 cases and 18.5% of ICD-10-only cases met SCID diagnostic criteria for an anxiety disorder in the 122–456 days time interval, and the relative odds were again not statistically significant (OR 1.16, 95% CI 0.55–2.46, *p* = 0.73). For the MINI, 49.2% of ICD-11 cases and 48.5% of ICD-10-only cases met MINI diagnostic criteria for a depressive disorder in the 0–60 days time interval and the relative odds were insignificant (OR 1.03, 95% CI 0.40–1.66, *p* = 1.00). In total, 45.4% of ICD-11 cases and 49.3% of ICD-10-only cases met MINI diagnostic criteria for an anxiety disorder in the 0–60 days time interval and the relative odds were again insignificant (OR 1.17, 95% CI 0.55–1.79, *p* = 0.637). However, ICD-11 cases had higher odds of a comorbid depressive or anxiety disorder diagnosis than ICD-10-only cases in the 122–456 days time interval (OR 2.34, 95% CI 1.16–4.76, *p* = 0.014 for depressive disorders; and OR 2.48, 95% CI 1.23–5.09, *p* = 0.009 for anxiety disorders); 65.9% of ICD-11 cases and 45.1% of ICD-10-only cases met MINI diagnostic criteria for a depressive disorder during this time interval. Similarly, 63.3% of ICD-11 cases and 40.9% of ICD-10-only cases met MINI diagnostic criteria for an anxiety disorder during this time interval.

### Studies’ heterogeneity

**Fig. 2.**
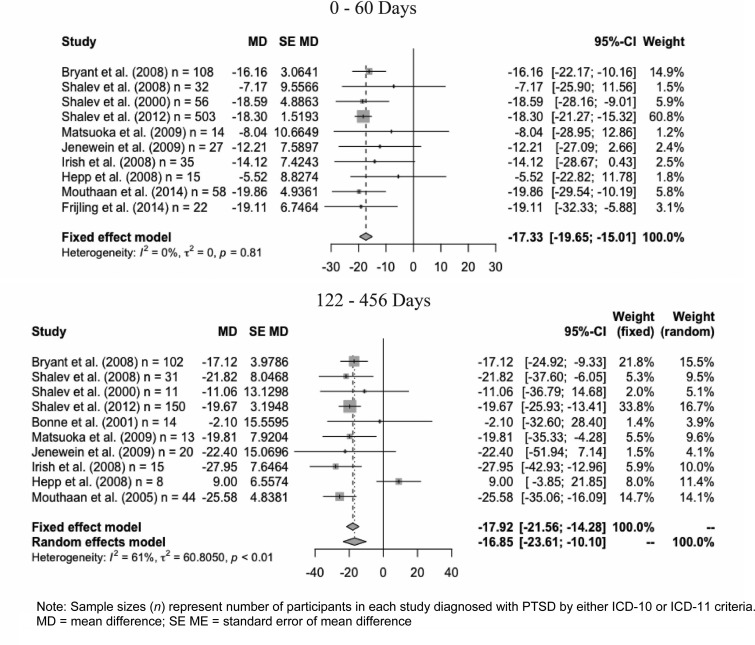
Studies’ heterogeneity at two time intervals.

A study-by-study analysis for the 0–60 days time interval, shown in Fig. 2, demonstrated a mixture of statistically significant and insignificant results, with no heterogeneity in terms of effects size and direction (*I*^2^ = 0%). Utilizing a fixed-effects model, an ICD-10-only classification had a mean decrease of 17.33 points on the CAPS total severity score relative to the ICD-11 (95% CI 15.01–19.65). The random-effects model's results were not different.

A study-by-study analysis (Fig. 2) for the 122–456 days time interval had moderate heterogeneity (*I*^2^ = 61%), with one study demonstrating a higher mean CAPS total severity score for the ICD-10-only group. However, utilizing a random-effects model, mean CAPS total severity score for the ICD-10-only group was still 16.85 points lower than the ICD-11 group's mean CAPS total severity score (95% CI 10.10–23.61).

## Discussion

The results of this study indicate that the ICD-11 consistently identifies fewer and more severe cases across time using both the more stringent and the more permissive re-experiencing criteria. Almost all the participants meeting ICD-11 PTSD diagnostic criteria also met ICD-10 criteria. About one-third to one-half of those meeting ICD-10 diagnostic criteria were not eligible for an ICD-11 diagnosis. They nonetheless reported moderate PTSD symptomology. These results were consistent after accounting for both the ICD-11 impairment and duration criteria, as well as potential heterogeneity of studies.

Both the disparity and the overlap in these data are consistent with the findings from previous literature examining the overlap in ICD-10 and ICD-11, e.g. 37.3% with ICD-10 PTSD *v.* 22.4% with ICD-11 PTSD in a sample of adolescents and young adults exposed to a mass shooting (Haravuori *et al.* 2016); 49.4% with ICD-10 PTSD *v.* 39.8% with ICD-11 PTSD among survivors of institutional abuse (Gluck *et al.* 2016); or 46.2% ICD-10 PTSD *v.* 33.7% ICD-11 PTSD in residents of conflict-afflicted Timor-Leste (Tay *et al.* 2017).

One explanation for the exclusion of ICD-10 cases by the ICD-11 may lie in the omission of several symptom criteria by ICD-11. In addition to excluding cognitive and affective symptoms previously recognized by both the ICD-10 and the DSM-5, the ICD-11 omits sleep disturbances and intense reactions to reminders (i.e. intense psychological distress at exposure to reminders, physiologic reactivity upon exposure to reminders without dissociative episode). These restrictions intentionally eliminate more diverse symptom presentations, effectively excluding PTSD cases previously identified under less stringent criteria.

Participants identified by the ICD-11 reported more severe symptoms than those identified by ICD-10. This finding is consistent with those of other studies: Both Gluck *et al.* (2016) and Haravuori *et al.* (2016) found that the cases identified by the ICD-11 were substantially more severe than those identified by the ICD-10. Importantly, participants identified by the ICD-10 criteria alone (i.e. excluded from ICD-11 diagnoses) had CAPS scores indicative of *moderate PTSD symptoms* and thus would require clinical attention (Weathers *et al.* 1999; Blake *et al.* 2000). Furthermore, participants identified by the ICD-10 alone had *the same or lower* likelihood of being diagnosed with a depressive or anxiety disorder, suggesting that alternative disorders do not account for the disparity in PTSD diagnoses. Using the ICD-11 to adjudicate treatment eligibility or coverage, therefore, might inappropriately restrict treatment access to many affected trauma survivors who may not be captured by other diagnoses. Though in line with ICD-11 declared goals of improving diagnostic specificity (Brewin, 2013; Maercker and Perkonigg, 2013), the resulting restriction warrants caution.

Observing participants’ PTSD classification over time revealed similar long-term outcome for both groups, expressed by similar rates of recovery and migration between diagnostic categories. Thus, the prognoses of those identified by ICD-11 and those identified by ICD-10 alone are relatively similar, the main difference lying in the severity of the individuals identified.

The use of the ICD-11 diagnostic criteria may also affect PTSD research, as they clearly restrict the group identified as having PTSD. This pattern of findings raises critical questions about the implications of the proposed ICD-11 definition of PTSD for research into this phenotype. In line with this idea, Stein *et al.* (2014) propose that restricting research to a narrowly defined phenotype (i.e. to either ICD-11, ICD-10, DSM-IV, or DSM-5 alone) is counterproductive because it presumes *a priori* assumptions of better homogeneity; they recommend that parsing research samples into homogenous subsets should follow empirical evidence optimally obtained by ‘broadly defined’ inclusion and subsequent testing of, among others, ICD-11 restrictions’ contribution to samples homogeneity.

This study was not free of limitations. First, the samples used in this study were not originally evaluated for ICD-10 or ICD-11 PTSD, and the measure used to derive these diagnoses was based on the DSM-IV template. At this point, however, items on the CAPS offer the best available approximation of ICD-10 and ICD-11 criteria, and in the absence of ICD-dedicated structured interviews, their use has been an unavoidable drawback across studies and recommendations to date (Brewin, 2013; Knefel and Lueger-Schuster, 2013; Gluck *et al.* 2016).

Second, this study used a 0–60 days time interval, resulting in very early CAPS assessments in many participants. ICD-11 criteria require that symptoms persist for several weeks. Consequently, assessment within the 0–60 days time interval, while indicative of early differences between ICD-10 and ICD-11 symptom templates, is not properly indicative of PTSD. However, we employed a 22–60 days time interval as a sensitivity analysis in order to examine the diagnostic rates of each ICD template while accounting for the minimum duration criterion and found consistent results regardless of the duration criterion.

Third, most of the study's participants were road traffic accident survivors, and others had undergone a single, salient event. The range of traumatic events studied may limit the generalizability of the study's results to other traumatic circumstances. Furthermore, measures of trauma severity, a difficult construct to measure, were not systematically administered and therefore were not included as a contributing factor.

Finally, the dataset used in this study did not include sufficient data to create variables representing diagnoses of complex PTSD (CPTSD), a sub-category of PTSD, or persistent grief disorder (PGD) as outlined by the ICD-11 (Maercker and Znoj, 2010; Cloitre *et al.* 2013; Maercker and Perkonigg, 2013). CPTSD criteria, however, require that all ICD-11 PTSD criteria be met, and therefore participants identified by ICD-10 alone would not have received CPTSD diagnosis. It is also unlikely that PGD would have compensated for the disparity between ICD-10 and ICD-11 diagnostic rates given that our sample represented mostly individuals who had experienced first-hand, non-interpersonal traumas.

The results of this study indicate that the proposed ICD-11 criteria do identify the more severe cases of PTSD. They nonetheless do not identify a substantial number of individuals with moderate symptoms who may not be captured or better classified by other diagnoses. As such, the use of ICD-11 criteria to sanction access to care should be considered cautiously, and their utility for research should be empirically validated. A clear advantage of the ICD template over the DSM routine is that its criteria are not as sharply positioned as essential determinants of construct boundaries and case identifiers, and thus leaving no space for medical expertise and experience. It would be, therefore, in the best ICD tradition to cautiously implement the new PTSD template and refrain from misusing it to define clinical caseness.
